# The medical competency training “climate-sensitive health counseling” – an interdisciplinary approach in planetary health education

**DOI:** 10.3205/zma001838

**Published:** 2026-04-15

**Authors:** Benedikt Lenzer, Raphael Kunisch, Elke Hertig, Verina Wild, Katharina-Jaqueline Wabnitz, Christoph Josef Schindler, Marco Roos

**Affiliations:** 1 Universität Augsburg, Medizinische Fakultät, Lehrstuhl für Allgemeinmedizin, Augsburg, Germany; 2 Universität Augsburg, Medizinische Fakultät, Regionaler Klimawandel und Gesundheit, Augsburg, Germany; 3 Universität Augsburg, Medizinische Fakultät, Institut für Ethik und Geschichte der Gesundheit in der Gesellschaft, Augsburg, Germany; 4 Universität Augsburg, Medizinische Fakultät, Department of Medical Education, Augsburg, Germany

**Keywords:** global health, planetary health, collaborative learning, medical education, global warming

## Abstract

**Objective:**

This article presents the Medical Competency Training (MCT) in Climate-Sensitive Health Counseling (CSHC) of the medical faculty Augsburg. The development and implementation process is described and reflected upon. In integrating Planetary Health Education into the medical curriculum at the University of Augsburg, an attempt was made to address its complex requirements through collaboration among various university institutes.

**Project description:**

Three university institutes developed the MCT CSHC. It comprises in-person sessions and e-learning and is integrated into the 8^th^ semester of the curriculum. The development process incorporated the chapter “planetary and global health” from the National Competency-Based Learning Objectives Catalog for Medicine, as well as criteria for high-quality planetary health education. The MCT CSHC imparts knowledge about environmental changes and their health consequences. It also fosters communication skills and an ethically reflected attitude.

**Results:**

The MCT CSHC was implemented from 2023 onwards. All characteristics of high-quality planetary health education, were incorporated into the MCT, with particular emphasis on developing transformative competencies, such as the ability to communicate in a climate-sensitive manner. The teaching materials were made publicly available. The MCT CSHC was further developed based on student and instructor feedback.

**Conclusion:**

The challenge of implementing a planetary health teaching format was met through the development and curricular integration of the MCT CSHC. Crucial to this success was the inclusion of three distinct institute perspectives on the scientific and practical topics arising within CSHC. A course evaluation to assess the learning objectives is planned.

## Zusammenfassung

**Zielsetzung:**

Dieser Beitrag stellt das *Ärztliche Kompetenztraining (ÆKT) Klimasensible Gesundheitsberatung (KSGB)* der medizinischen Fakultät Augsburg vor. Dabei wird der Entwicklungs- und Implementierungsprozess dargestellt und reflektiert. Es wurde versucht, den komplexen Anforderungen der Planetary Health-Lehre durch Zusammenarbeit verschiedener Institute bei der Integration in das medizinische Curriculum der Universität Augsburg zu begegnen.

**Projektbeschreibung:**

Das ÆKT KSGB wurde von drei Lehrstühlen entwickelt. Es umfasst Präsenz-Einheiten sowie E-Learning und ist im 8. Semester des Curriculums verankert. In die Entwicklung ist das Kapitel Planetare und Globale Gesundheit des Nationalen Kompetenzbasierten Lernzielkatalogs Medizin sowie Kriterien für qualitative Planetary Health-Lehre eingeflossen. Im ÆKT KSGB wird Wissen zu den Umweltveränderungen und den gesundheitlichen Folgen vermittelt. Es wird Kommunikationskompetenz und eine ethisch reflektierte Haltung vermittelt.

**Ergebnisse:**

Das ÆKT KSGB konnte ab 2023 durchgeführt werden. Alle Charakteristika qualitativer Planetary Health-Lehre. wurden im ÆKT umgesetzt, wobei auf die Ausbildung transformativer Kompetenzen, wie die Fähigkeit klimasensibel zu kommunizieren, Wert gelegt wurde. Die Lehrmaterialien wurden öffentlich bereitgestellt. Das ÆKT KSGB wurde aufgrund von Rückmeldung der Studierenden und Lehrenden weiterentwickelt.

**Schlussfolgerung:**

Der Herausforderung, ein komplexes Planetary Health-Lehrformat zu verwirklichen, konnte durch den Aufbau des ÆKT KSGB mit curricularer Verankerung begegnet werden. Entscheidend waren hierbei die Einbringung drei verschiedener Institutsperspektiven zu den Wissenschafts- und Praxisthemen, die sich im Rahmen der KSGB ergeben. Eine Kursevaluation zur Überprüfung der Lernziele wird angestrebt.

## 1. Introduction

In light of the climate crisis and further exacerbating ecological crises, such as the biodiversity crisis, there has been an increase in teaching projects on planetary health in Germany [1], [2], [3]. In 2023, an editorial in the GMS Journal of Medical Education summarized: “The future mental and physical health of people depends on overcoming the climate crisis. It’s that simple. It’s that complex.” ([4], lines 1-3). Shaw et al. describe Planetary Health Education (PHE) as a process of equipping medical personnel with the knowledge, skills, and values needed to be able to act and speak accordingly. Furthermore, in light of the multiple ecological and social crises, self-efficacy should be fostered to achieve the necessary transformation towards planetary health [5]. Medical professionals should actively support this transformation due to their roles in society, patient care, teaching, and research [6]. Therefore, teaching formats are needed that impart the necessary knowledge and skills as well as contemporary attitudes for the difficult task of addressing environmental changes and their health consequences in everyday clinical practice [7]. To our knowledge, there are currently only a few teaching concepts that convey planetary health in an interdisciplinary manner, from basic scientific findings and clinical medicine to the socio-ethical dimension. Interesting examples include a “longitudinal mosaic curriculum on planetary health” implemented at the Würzburg faculty and an elective course “climate consultation” at the Universities of Giessen and Marburg [8], [9]. In Würzburg, efforts are being made to integrate planetary health into the curricula of all disciplines across the semesters, which is universally welcomed for its relevance, but is very time-consuming due to the need to coordinate with the various instructors. Accordingly, it becomes apparent in the elective subject that, due to the lack of curricular integration, only a small proportion of students are reached. Simon et al. described ten characteristics for successful PHE [10]:

1. Complexity and systems thinking,
2. Inter- and transdisciplinarity,
3. Ethical dimensions,
4. Responsibility of healthcare professionals,
5. Transformative competencies including practical skills,
6. Space for reflection and resilience building,
7. Special role of students,
8. Curricular integration,
9. Innovative and recognized teaching methods,
10. Education as a driver of innovation.

The characteristics represent a synthesis of the results of a qualitative interview study and the evaluation of three international frameworks for PHE (see [5], [11], [12]). The relevance of the criteria for PHE can be illustrated using the characteristic of complexity and systems thinking: Environmental changes lead to health consequences, which are further influenced by factors such as heat exposure in urban areas. Therefore, medical solutions must take into account the social and political context of the health problem. These characteristics demonstrate how comprehensively the topic must be considered in the design of teaching sessions and in curricular integration. However, at the level of individual faculties, medical curricula often appear rigid and already overloaded, making it challenging to implement a cross-departmental teaching program [13]. This is significant because the cross-cutting topic of planetary health touches upon all medical disciplines and requires interdisciplinarity [2]. This article introduces the process and structure of the Medical Competency Training (MCT) in Climate-Sensitive Health Counseling (CSHC). It also describes how the various requirements of PHE were addressed during its integration into the medical curriculum at the University of Augsburg, and how, in particular, the collaboration between different institutes played a role in its development and implementation.

## 2. Project description

### 2.1. Development of the MCT CSHC

The MCT was developed under the leadership of the institutes of general medicine and ethics and history of health in society, in close cooperation with the chair of regional climate change and health, for implementation within the Augsburg curriculum. It consists of four in-person sessions and an individual e-learning phase. 

This teaching concept introduces the concept of CSHC [14], [15]. Medical students apply their knowledge of the relationship between the health effects of human-induced environmental influences/burdens and individual lifestyles. The skills to be acquired include communication abilities and the capacity to provide substantive advice. Furthermore, the students should develop an ethically reflected attitude. Working in groups, the students create a communication product (e.g., flyer, waiting room poster) which is then made available to medical practices. The development of this MCT was carried out incorporating the National Competency-Based Learning Objectives Catalog for Medicine, specifically the chapter planetary and global health [16]. The teaching concept, including the teaching and learning materials, was reviewed by the participating institutes before implementation. Lecture notes, including flowcharts, were prepared for the instructors in accordance with the AVIVA principle [17]. The criteria catalog by Simon et al. was used to revise and improve the course [10]. The developed communication products were made available to interested physicians via the German society for general practice and family medicine and the Augsburg teaching practice network. The institute of general practice was responsible for the overall organization, student support, and provision of teaching materials during the development and implementation phases. After each teaching session, a lecturer’s reflection form was completed and analyzed by the teaching management team of the chair of general practice. This form inquired, among other things, whether the interactive methods were effective and what changes were desired. The suggestions were incorporated into the second iteration of the MCT program in the summer semester of 2024. The full teaching materials including the instructor scripts were uploaded to the virtual Planetary Health Education Toolbox ([https://openwuecampus.uni-wuerzburg.de/moodle/mod/folder/view.php?id=10240&lang=en], 03.08.2025).

### 2.2. Integration into the curriculum

**Figure 1:**
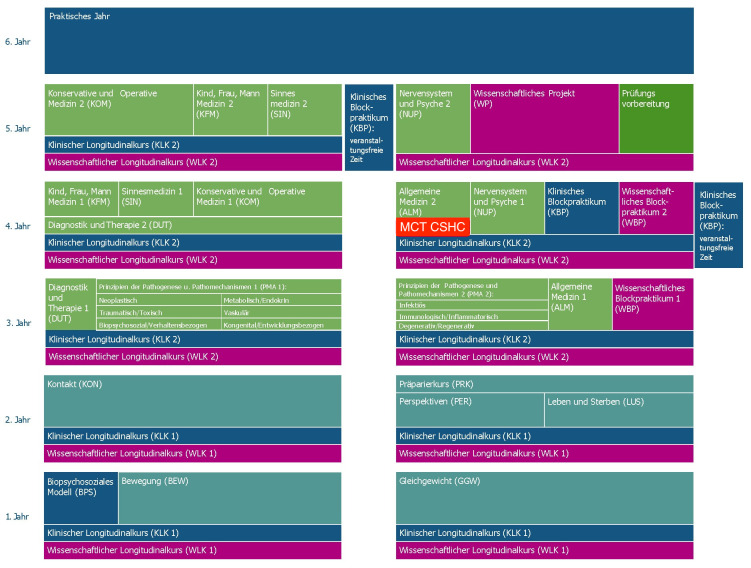
Integration of the MCT CSHC into the curriculum of the medical degree program (only in German)

The model medical degree program at the University of Augsburg started in the winter semester of 2019/20. Teaching takes place in organ- and system-oriented modules (horizontal integration) with increasing complexity throughout the course of study (vertical integration) [18]. Clinical and scientific competencies are imparted through longitudinal courses. Planetary health is integrated into both, the core and elective curriculum. Ethics and communication are emphasized in the first years of study but are taught throughout the degree. A range of MCTs on diverse topics such as incidental findings are delivered over the course of the degree (see figure 1 (Fig. 1)).

Graduates should not only possess biomedical knowledge but also acquire corresponding practical skills [19]. Therefore, in the MCTs within the compulsory curriculum, students work on everyday medical tasks and produce concrete products (medical case reports, pocket guides for the first night shift, etc.) in a project- and team-based manner. MCTs are interdisciplinarily and professionally oriented, aligned with the entrustable professional activities (EPA) framework.

Students work and learn collaboratively and individually, situating theory in practice and developing metacognitive strategies highly relevant to future clinical practice. Instructors provide professional advice and guidance.

The concept of MCTs expands upon existing competency-oriented teaching formats such as case discussions or bedside teaching by addressing medical situations in a broader context than is possible in individual courses [20]. The MCT CSHC was first implemented in the clinical longitudinal course during the 8th semester in 2023.

### 2.3. MCT CSHC process and exemplary learning objectives

#### 2.3.1. Introduction

**Figure 2:**
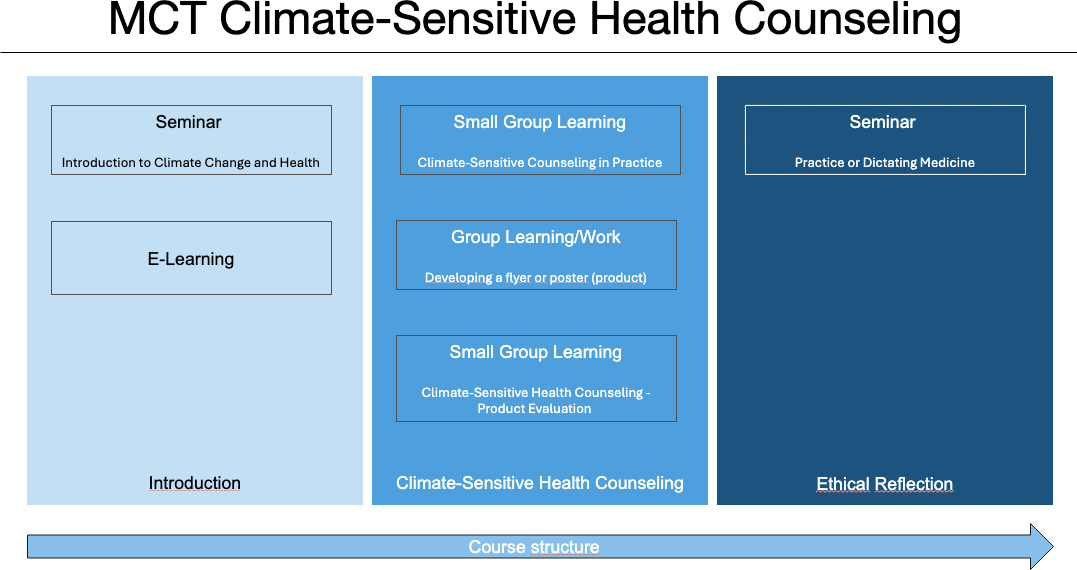
Course structure

In the seminar “introduction to climate change and health” (chair of regional climate change and health, one session of 45 minutes, social form: plenary lecture and interactive), the basic knowledge of planetary boundaries, the doughnut economy, anthropogenic climate change and its effects on health are explained (see figure 2 (Fig. 2)). Students are introduced to the concept of co-benefits. Co-benefits arise when climate-friendly and simultaneously health-promoting measures are implemented [21]. This could include lifestyle changes towards more active, sustainable mobility, or regional and seasonal food consumption and the reduction of animal products. Important learning objectives encompass understanding the connections between climate change and health, as well as the planetary boundaries. Using the example of co-benefits, students can reflect on the complexity of the relationship between the environment and health, as well as its links to other levels such as politics, society, and economics. The e-learning platform offers relevant foundational articles. These publications cover the concept of planetary health and co-benefits, the CSCH , including its underlying moral stance, communication skills, and medical ethos in times of ecological crises [14], [15], [20], [21], [22], [23], [24], [25], [26], ([https://www.wma.net/policies-post/wma-international-code-of-medical-ethics/], 19.03.2025). Furthermore, a PowerPoint presentation with voice-over is offered for further study. It explains to students the emergence and development of the concept of CSHC in research and practice. It then includes a classification and explanation of the conceptual foundations of CSHC, including appropriate methods, necessary prerequisites, and reasons for consultation, along with the corresponding content and topics. Here too, co-benefits are revisited and illustrated with examples from everyday general medical practice.

#### 2.3.2. Climate-sensitive health counseling

**Figure 3:**
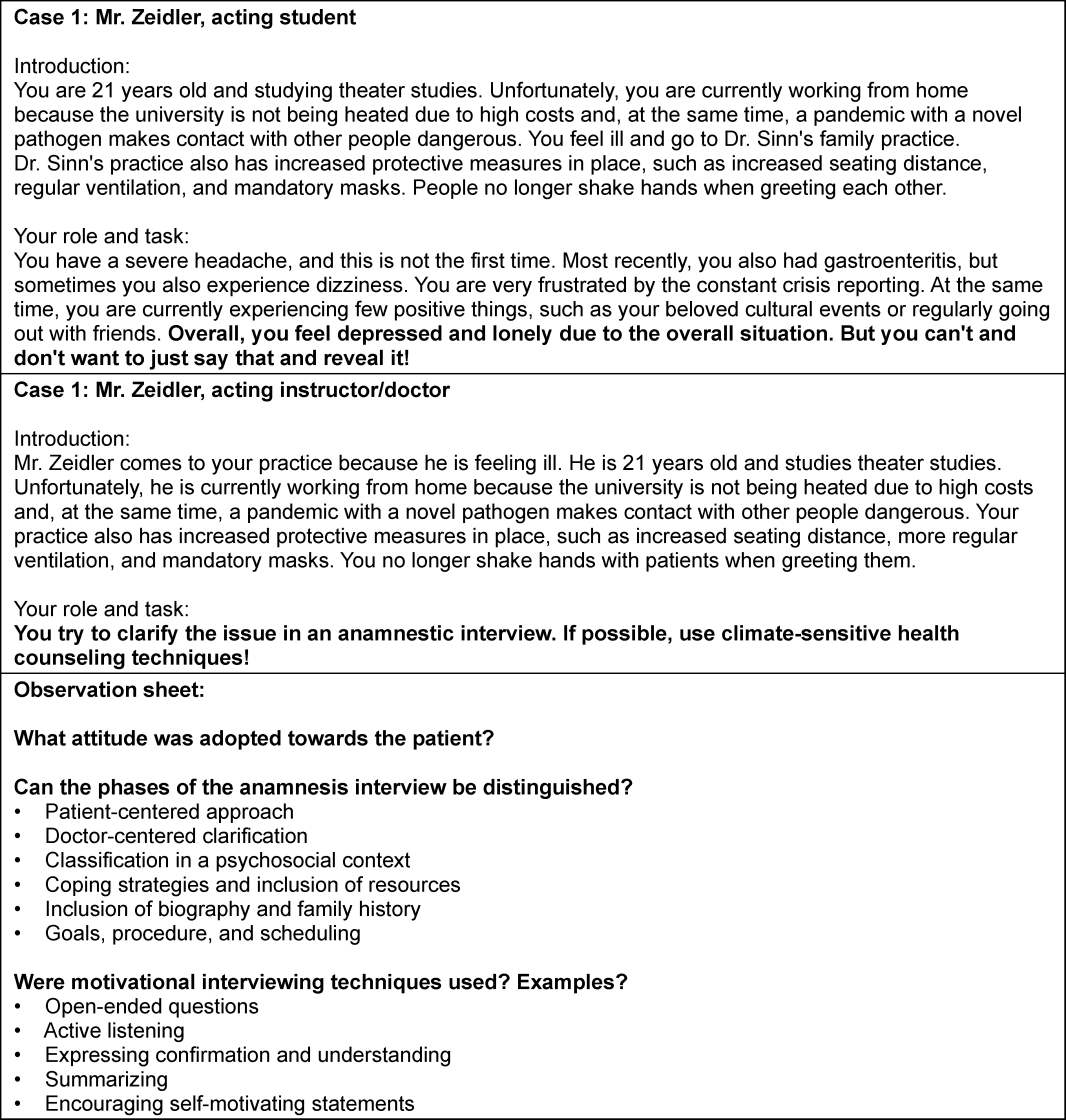
Example of a case vignette including observation sheet

In the small group format “climate-sensitive counseling in practice” (chair of general medicine, two sessions of 45 minutes), the social format alternates between plenary lectures and interactive sessions. Interactive work is carried out in conversation simulations and discussion rounds. The knowledge acquired so far is reflected upon, students’ prior experiences are discussed, and potential pitfalls of CSHC in general practice are highlighted. The instructors can share tips and experiences from their own daily practice. Furthermore, communication skills focused on motivational interviewing are practiced through role-playing exercises (e.g., the case vignette in figure 3 (Fig. 3)). This also targets the ability to structure conversations, which is vital for CSHC. After a reflection on the sessions, the group work phase concludes with instructions for developing the product. Important learning objectives of these sessions include the ability to describe the concept of Planetary Health and to formulate co-benefits. It also aims to improve motivational interviewing skills. 

**Figure 4:**
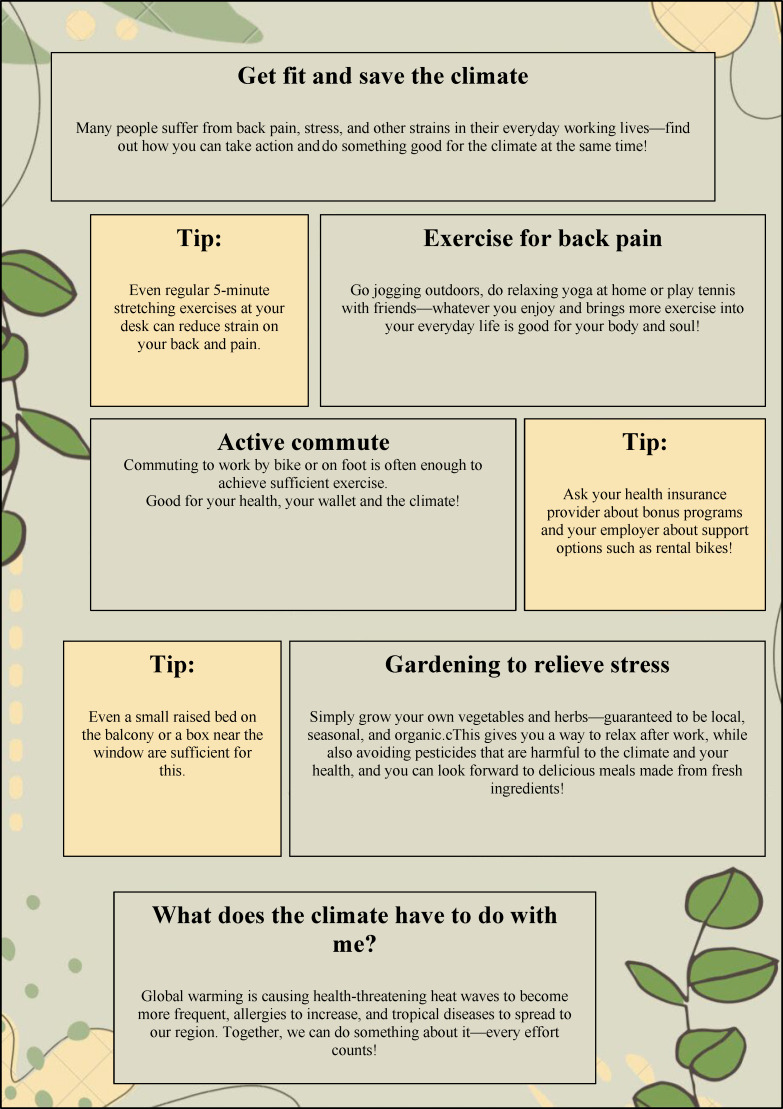
Example of a flyer designed by students

The students in the small group organize themselves to create a healthcare-related communication product and can work digitally or in person. Up to six sessions of 45 minutes each are allocated in the timetable of the curriculum for this purpose. A case vignette illustrates a healthcare or problem situation in a general practitioner's office. To solve the problem, students are asked to create a communication product collaboratively. For example, they are to create a flyer explaining the planetary health diet to unburden the limited consultation time (see figure 4 (Fig. 4)). All resources, including the use of artificial intelligence, are permitted for this task. The purpose of this session is to apply CSHC to a product actually used in general practices, employing motivational communication techniques.

In the following small group activity climate-sensitive health counseling – product evaluation (chair of general medicine, two sessions of 45 minutes each), the social forms of plenary lectures and interactive sessions are used. In a “crossover” approach, the students conduct a peer review of another small group’s product (see figure 4 (Fig. 4)). The purpose of this small group activity is to stimulate reflection on the development process of their own product. Based on this, recommendations for product improvement are formulated in a reciprocal manner.

#### 2.3.3. Ethical reflection

The MCT concludes with the seminar “practice or dictating medicine” (one session of 45 minutes), delivered by the institutes for general medicine and ethics and history of health in society. The seminar is structured around the social formats plenary sessions (both frontal and interactive) and group sessions (both frontal and interactive). The four corners method is employed [27]. Reflection and discussion are guided by the following questions to approach the complex ethical considerations (see [6]):

1. Climate change and health – What aspects of it overwhelm me? What do I find difficult?
2. How can I shape the doctor-patient relationship?
3. What can I do in my practice?
4. What can I do in terms of professional policy?

In small groups, students discuss the practical, emotional, and moral challenges associated with climate- and environmentally conscious medical practice. They develop strategies for fulfilling their increasing professional responsibilities. In doing so, they also learn to recognize their own, institutional, and structural limitations and to find constructive ways to address them. A final plenary session addresses the challenges and potential solutions. The overarching learning objective is to develop an ethically reflected professional attitude in times of human-induced environmental changes.

## 3. Results

### 3.1. Development and Implementation

The MCT CSHC was developed within six months in collaboration of the participating institutes. Due to the strong motivation of those creating the MCT CSHC, as well as their extensive expertise in their respective fields, the development could be carried out in a decentralized, asynchronous manner. No in-person planning meetings were held. Necessary communication was handled via email, Microsoft Teams, and individual arrangements. The schedule and structure of the course were developed by the heads of the participating institutes in consultation with the dean’s office and the department of medical education. The content development was undertaken by staff members with relevant experience in PHE. This demonstrated that the interdisciplinary approach provided valuable input, particularly in designing the schedule and coordinating the sessions of the MCT CSHC. The feedback received during the mutual review of the teaching materials focused primarily on formal points. Following the publication of the criteria catalog by Simon et al., the lecturers’ scripts were reviewed to identify areas for improvement. The results highlighted the importance of creating a healthcare-relevant communication product, as the findings are actually published for use in medical practices. Furthermore, the transdisciplinary connections involved in translating planetary health into practice were made more explicit in the sessions delivered by the institute for general practice. The MCT CSHS has so far been conducted during the summer semesters of 2023-2025. Students created 11 flyers, mainly with the help of artificial intelligence. We consider the content and graphic quality to be high. During the MCT CSHC, fluctuating participation was observed, particularly at off-peak times or near semester exams, as attendance was not mandatory. This often complicated interactive elements such as the role-playing exercises, as only a small number of students were present. It was observed that these students, who were already particularly interested in the topic, sometimes reacted strongly emotionally during the ethical reflections.

Currently, no systematic semester evaluations are available to draw conclusions regarding further development of the MCT CSHC course. However, a curriculum-wide evaluation is planned. Individual feedback conversations between lecturers and students have yielded positive verbal feedback on the course. However, the structure with overlapping online and in-person sessions, as well as the timetable design, were deemed problematic. In some cases, the timeframes for creating a healthcare-related communication product were too tight. Lecturers observed that the relevance of in-person sessions for students strongly depends on their usefulness in graded exams. Student feedback was used to improve the presentation of the MCT CSHC course and group work on the e-learning platform Moodle and to make the online sessions more visible in the timetable. During implementation, the usefulness of the existing lecturer scripts became apparent in compensating for staff absences. These were adapted and shortened over the course of the terms. The need for a designated person to coordinate the lecturers and serve as a point of contact for students became apparent.

### 3.2. Qualitative planetary health teaching

According to the authors, all 10 characteristics, including the implementation of inter- and transdisciplinarity and the ethical dimension, could be addressed [8]. For better comprehension, we provide a detailed description of the characteristics with examples from the MCT CSHC in the attachment 1 .

## 4. Discussion

In 2023, the medical compentency training climate-sensitive health counseling was implemented for the first time at the faculty of medicine of the University of Augsburg. The challenge of providing high-quality PHE was met through the hybrid structure of the course. Crucial to this was the collaboration of three institutes to cover the scientific and practical topics arising from climate-sensitive health counseling (general medicine, regional climate change and health, ethics and history of health in society). This was complemented by reflective analysis of the acquired knowledge and skills, as well as by addressing the emotional needs of the students through professional ethical guidance and by lecturers with practical experience [7]. Initial experiences showed that some students developed a strong emotional involvement, even feeling powerless. This led to consideration of whether these students should be offered support beyond the existing courses, or whether the final reflection session incorporating ethics should be expanded with an additional preparatory session at the beginning of the MCT. The MCT concept appears to be particularly well-suited for teaching Planetary Health, as it places students at the center and thus acknowledges their role in the ecological transformation of society and medicine [10]. By applying the criteria for high quality PHE, course content could be further refined, and the entire course underwent a comprehensive review. The implementation of such a comprehensive Planetary Health teaching project was particularly successful because the Augsburg curriculum allocates a significant number of teaching hours [14] to this topic at the participating departments and promotes interdisciplinary teaching. However, this also implies that such a high number of hours is not feasible at all universities. Therefore, alternative options should be explored, such as creating elective courses [13], [28]. The process also presents challenges for students and instructors, such as difficulties in creating timetables. It remains unclear which factors influence student participation in planetary health courses. However, the need to teach planetary health will persist; therefore, efforts to integrate it into the curriculum should be expanded across other faculties as well. Specific open questions that should be addressed through a course evaluation and methodological support of the MCT CSHC are:

1. Is the MCT effective in imparting knowledge and skills related to planetary health?
2. Does the MCT foster a reflected attitude?
3. How does the professional identity of students change through their engagement with planetary health and CSHC in practice?

Furthermore, it would be interesting to learn how sustainably the acquired content and skills can be integrated into further student and later medical practice.

## 5. Conclusion

The MCT CSHC project demonstrates that a comprehensive planetary health course is feasible and that interdisciplinary networking supports high-quality PHE. The presented requirements for good PHE highlight the importance of curricular integration and sufficient teaching hours. The faculty’s focus on environmental health sciences facilitated the involvement of institutes with a connection to planetary health. The project can serve as an example and inspiration for the implementation or further development of PHE in Augsburg and beyond. Naturally, the results of the planned evaluations must be considered, and the MCT CSHC project should be continuously developed.

## Funding

This work was partially funded by the seed funding project “environmental health ethics and justice”, grant number 2023-13, of the University of Augsburg.

## Authors’ ORCIDs

- Benedikt Lenzer: [0000-0003-2239-797X]
- Raphael Kunisch: [0000-0002-0758-5721]
- Elke Hertig: [0000-0002-6934-9468]
- Verina Wild: [0000-0003-3012-7662]
- Katharina-Jaqueline Wabnitz: [0000-0002-2394-101X]
- Christoph Josef Schindler: [0009-0009-7131-4422]
- Marco Roos: [0000-0003-1596-5908]

## Competing interests

The authors declare that they have no competing interests. 

## Supplementary Material

The medical competency training “climate-sensitive health counseling” – an interdisciplinary approach in planetary health education
