# Supplementary material for: The medical competency training “climate-sensitive health counseling” – an interdisciplinary approach in planetary health education
Source: GMS J Med Educ. 2026 Apr 15;43(4):Doc44. doi: 10.3205/zma001838 (PMC13124464; doi:10.3205/zma001838)
Supplement: The medical competency training “climate-sensitive health counseling” – an interdisciplinary approach in planetary health education [file JME-43-44-s-001.pdf]

Attachment 1: The medical competency training “climate-sensitive health counseling” – an interdisciplinary approach in planetary health education

1. Complexity and systems thinking

This characteristic is reflected in the thematic and longitudinal intertwining of knowledge acquisition and competence development within the four face-to-face sessions and in the phases of individual learning. The complexity of the content, including references to a wide range of disciplines, is reflected in the knowledge transfer, but also in the interrelationships between the individual teaching units, which are organized by different departments. Systemic thinking is trained through the integration of what has been learned in the development of the communication product and ethical reflection. The content is embedded in the curriculum of the medical program, which also emphasizes the importance of processing complex issues through systemic thinking.

2. Inter- and transdisciplinarity

The MCT is organized and run by three institutes/departments: General medicine, regional climate change and health, and ethics and history of health in society. This approach promotes interdisciplinarity. This way of working is common practice at the University of Augsburg’s faculty of medicine, which enriches teaching and research and also contributes to smooth implementation.

3. Ethical dimensions

The courses continuously address ethical issues in the context of environmental and climate change, health consequences and inequalities, resource consumption through medical care, and the role of physicians themselves. The question of the individual right of patients to the best possible care and the right of society as a whole to resource-efficient healthcare is examined. In particular, co-benefits are highlighted as a way of better meeting the demands of good healthcare and resource conservation. As a special feature of the MCT, a final teaching unit is dedicated specifically to ethical issues and possible courses of action.

This topic is explicitly addressed in the units climate-sensitive counseling in practice and practice or dictating medicine. Beyond the MCT, these topics are covered in detail in the teaching units on general medicine, ethics and history of medicine, and medical psychology/communication, allowing the MCT to build on this knowledge and these skills.

Attachment 1 to Lenzer B, Kunisch R, Hertig E, Wild V, Wabnitz K, Schindler C, Roos M. *The medical competency training “climate-sensitive health counseling” – an interdisciplinary approach in planetary health education.* GMS J Med Educ. 2026;43(4):Doc44. DOI: 10.3205/zma001838

4. Transformative competencies including practical skills

Since the MCT aims not only to impart basic knowledge but also to develop communication skills for KSGB and to encourage a contemporary, reflective attitude in times of climate and environmental change, this characteristic is fulfilled. The practical skills include communication skills and counseling using creative methods, including the creation of a graphic communication product.

5. Space for reflection and resilience building

The MCT offers students plenty of room for reflection, as the four classroom sessions build on each other. Reflection is also facilitated by the discursive nature of the teaching units. We believe that supervised reflection and the development of an attitude guided by planetary health values, together with practical skills, will promote students' resilience in the face of growing environmental crises.

6. Special role of students

The students' scope for action increases over the course of the MCT, which should lead to an improvement in their decision-making and action-taking skills. Thus, over the course of the MCT, the role of the students changes from learners to active participants through the creation of the communication product and to reflective actors who also reconsider their own limitations and possibilities. Due to the high proportion of reflection throughout the MCT, teachers often take on a mentoring or supervisory role.

7. Curricular integration

The MCT is fully integrated into the medical curriculum. The model degree program makes it particularly easy to build on skills and content from previous years of study and to further develop newly acquired knowledge in later years.

8. Innovative and recognized teaching methods

During the development of the MCT, modern medical teaching methods were implemented thanks to the high level of medical teaching qualifications of the participating teaching staff and the technical expertise of the department of medical education Augsburg. Among other things, the Munich method box was used to select the methods.

9. Education as a driver of innovation

Through the exemplary application of CSHC in the creation and dissemination of the communication product, we assume that students will develop the competence to respond to new problems (e.g., additional need for counseling due to environmental changes) with creative methods (e.g., AI-supported generation of information material). At the same time, we hope to spread the innovative potential of CSHC by distributing the communication products via the teaching practice network of the institute of general medicine.

10. Education as a driver of innovation

Through the exemplary application of CSHC in the creation and dissemination of the communication product, we assume that students will develop the competence to respond to new problems (e.g., additional need for counseling due to environmental changes) with creative methods (e.g., AI-supported generation of information material). At the same time, we hope to spread the innovative potential of CSHC by distributing the communication products via the teaching practice network of the institute of general medicine.

Attachment 1 to Lenzer B, Kunisch R, Hertig E, Wild V, Wabnitz K, Schindler C, Roos M. *The medical competency training "climate-sensitive health counseling" – an interdisciplinary approach in planetary health education.* GMS J Med Educ. 2026;43(4):Doc44. DOI: 10.3205/zma001838
